# Tridimensional model structure and patterns of molecular evolution of *Pepino mosaic virus *TGBp3 protein

**DOI:** 10.1186/1743-422X-8-318

**Published:** 2011-06-24

**Authors:** Beata Hasiów-Jaroszewska, Anna Czerwoniec, Henryk Pospieszny, Santiago F Elena

**Affiliations:** 1 Institute of Plant Protection-National Research Institute, ul. Wł. Węgorka 20, 60-318 Poznań, Poland; 2 Bioinformatics Laboratory, Institute of Molecular Biology and Biotechnology, Adam Mickiewicz University, Umultowska 89, PL-61-614 Poznan, Poland; 3 Instituto de Biología Molecular y Celular de Plantas, CSIC-UPV, 46022 València, Spain; 4 The Santa Fe Institute, Santa Fe, NM87501, USA

**Keywords:** molecular evolution, PepMV, protein modeling, selective constraints, TGBp3, virus evolution

## Abstract

**Background:**

*Pepino mosaic virus *(PepMV) is considered one of the most dangerous pathogens infecting tomatoes worldwide. The virus is highly diverse and four distinct genotypes, as well as inter-strain recombinants, have already been described. The isolates display a wide range on symptoms on infected plant species, ranging from mild mosaic to severe necrosis. However, little is known about the mechanisms and pattern of PepMV molecular evolution and about the role of individual proteins in host-pathogen interactions.

**Methods:**

The nucleotide sequences of the triple gene block 3 (TGB3) from PepMV isolates varying in symptomatology and geographic origin have been analyzed. The modes and patterns of molecular evolution of the TGBp3 protein were investigated by evaluating the selective constraints to which particular amino acid residues have been subjected during the course of diversification. The tridimensional structure of TGBp3 protein has been modeled *de novo *using the Rosetta algorithm. The correlation between symptoms development and location of specific amino acids residues was analyzed.

**Results:**

The results have shown that TGBp3 has been evolving mainly under the action of purifying selection operating on several amino acid sites, thus highlighting its functional role during PepMV infection. Interestingly, amino acid 67, which has been previously shown to be a necrosis determinant, was found to be under positive selection.

**Conclusions:**

Identification of diverse selection events in TGB3p3 will help unraveling its biological functions and is essential to an understanding of the evolutionary constraints exerted on the *Potexvirus *genome. The estimated tridimensional structure of TGBp3 will serve as a platform for further sequence, structural and function analysis and will stimulate new experimental advances.

## Background

*Pepino mosaic virus *(PepMV) belongs to *Potexvirus *genus within the *Flexiviridae *family and it is a well-known pathogen infecting tomato plants worldwide. PepMV possesses a single-stranded, positive-sense RNA genome of approximately 6.4 kb, flanked by 5' and 3' untranslated regions (UTRs) with a 5' cap and a 3'poly(A) tail. PepMV contains five conserved open reading frames (ORFs). ORF1 encodes a 164 kDa replication-related protein with three functional domains: an N-terminal mRNA capping enzyme, a central RNA helicase, and a C-terminal RNA-dependent RNA polymerase (RdRp). ORF2 through ORF4 encode movement proteins (TGBp1, TGBp2 and TGBp3) of 26, 14, and 9 kDa, respectively. These three ORFs overlap in the genome and, hence, are called the triple gene block. The 25 kDa CP is encoded by ORF5 [1-3]. The virus is highly diverse; four different genotypes (CH2, LP, EU, and LP) and a large number of inter-strain recombinants have been described [4,5]. PepMV isolates differ both in host range as well in the symptoms induced on susceptible plants species. Recently, it was proved that one single mutation in TGBp3 protein converts a mild pathotype of CH2 genotype into a necrotic one. This is the only association reported between particular amino acid sites and symptomatology [6]. However, our preliminary results with isolates from the EU genotype showed that we are dealing with a universal mechanism of variability. By performing site-directed mutagenesis of infectious clones of the EU genotype, we confirmed that amino acid 67 is responsible for inducing necrosis on tomato, irrespective of the strain (unpublished results). This finding suggests that TGBp3 structure is important for virulence, since substitution of certain amino acids results in changes in the infectivity of the virus. As the tridimensional structure of TGBp3 is not yet available, *de novo *modeling represents a suitable and convenient alternative for analyzing the relationship between amino acids present at certain positions, structure and virulence. This is the approach undertaken in this study.

A second aim of this study is to verify the nature of the selective pressures operating on TGBp3. Nucleotides substitutions can be classified into two categories on the basis of their effects on the protein amino acid composition: synonymous and nonsynonymous replacements. In general synonymous substitutions accumulate at rate *d*_*S *_per synonymous site. It is generally accepted that synonymous substitutions accumulate neutrally because they have no effect on the amino acids composition and thus shall not affect the folding and function of proteins. In contrast, nonsynonymous substitutions accumulate at rate *d*_*N *_per synonymous site. Since these substitutions involve amino acid replacements, they must be subjected to selection. The average ratio of these two substitutions rates *ω *= *d*_*N*_/*d*_*S *_across a gene, provides information on whether it has been fixing amino acid replacements in a neutral fashion (*ω *= 1), amino acids changes have been removed by the action of purifying selection (*ω *< 1), or changes have been fixed by positive evolution (*ω *> 1) [7]. However, averaging *ω *across the entire coding sequence of a gene is a gross approximation since highly conserved and hypervariable sites may coexist. It is more sensitive to allow the *ω *rates ratio to vary among sites and to take the presence of individual sites at which *ω *> 1 as an evidence for positive selection [7]. In this study, sequence analysis of the PepMV TGB3 was conducted and *ω *rates ratio were calculated. Additionally, a computational analysis of TGB3 sequences to investigate the extent of recombination events was performed. Taken together, in this study we present results of computational analyses evaluating the patterns of molecular evolution and their effect on protein structure and virulence.

## Methods

### Virus isolates and phylogenetic analysis

Eighteen sequences of PepMV TGB3 representing isolates from different genotypes and geographic regions were retrieved from GenBank and used for further analysis (Table 1). For some isolates detailed biological information was also available. Sequences were translated into amino acids using BioEdit [8]. Multiple protein sequence alignments were obtained using MUSCLE [9] and then protein-coding nucleotide sequence alignments were constructed based on their corresponding protein sequence alignments using PAL2NAL[10].

**Table 1:** Isolates used in study

Isolate	Origin	Genotype	Symptoms on *S. lycopersicum*	Accession Numbers
1806	Belgium	EU	mild	FJ457098
DB1	Netherlands	EU	necrosis	FJ940224
SW	Poland	EU	mild	EF408822
UK	UK	EU	-	AF340024
SP13	Spain	EU	-	AF484251
PMUO618	Spain	EU		FJ263318
H	Hungary	EU	mild (yellow mosaic)	AM491606
No	Poland	EU	mild	FJ612602
LP2001	Peru	LP	asymptomatic	AJ606361
US3	USA	EU	-	AY508411.1
Ch1	Chile	CH1	-	DQ000984.1
P22	Poland	CH2	mild	HQ650560
Cl308	Spain	CH2	-	GU130097
PMU0848	Spain	CH2	-	FJ263365
P19	Poland	CH2	necrosis	HQ650559
Ch2	Chile	CH2	-	DQ000985
Amo7	Spain	CH2	-	GU130090
US2	USA	recombinant CH1/CH2	-	AY509927

It is well known that recombination affects the estimation of *ω *and interferes with phylogenetic reconstruction [11]. Therefore the PepMV sequences were initially analyzed for evidence of recombination events using the RDP package [12]. The sequences were analyzed using the following methods: 3 Seq, Chimaera, Lard, Bootscan, RDP, Genecovn, and MaxChi. Only recombination breakpoints supported by more than three methods were considered as valid. With this stringent criterion, no significant recombination signals were identified in the analyzed sequences. The best-fitting model of nucleotide substitution was investigated using the MODELTEST implemented in MEGA5; Kimura 2-parameters model (K2P) was chosen. Maximum-likelihood phylogenetic tree was constructed using MEGA5 and K2P model of nucleotide substitution [13]. Reliability of the obtained tree was evaluated using the bootstrap method based on 1000 pseudoreplicates. Both nucleotide and amino acids sequences were scrutinized to find particular positions distinguishing mild and severe isolates.

### Analysis of selective pressure

Our analysis of selective pressures was based on evaluating *ω *for each codon in the nucleotide sequence alignment. The single-likelihood ancestor counting (SLAC), fixed-effects likelihood (FEL) and internal branches fixed-effects likelihood (IFEL) methods were used to evaluate *ω *per codon [14]. Both SLAC and FEL methods used using the default significance level of *p *= 0.1; a Bayes factor of 40 was used as selection threshold for REL. These analyses were performed using the HyPhy package [15] as implemented in the DATAMONKEY webserver [16]. Nucleotide substitutions were modeled according to the K2P scheme. If a gene evolves under purifying selection most of the time but is occasionally subject to episodes of positive selection, a comparison between two distant related sequences is unlikely to yield a *ω *ratio significantly > 1. To specifically test whether phylogenetic branches with necrotic isolates underwent positive selection events, we performed two independent analyses of branch-specific codon models. First, the M2 branch site model allowing *ω *to vary among sites in the protein and across branches on the tree and aim to detect positive selection affecting a few sites along particular lineages [17] was evaluated using the program CODEML from the PAML version 4.4 package [18]. Second, we applied the sliding-window analysis implemented in SWAPSC [19]. SWAPSC uses a statistically optimized window size to detect selective constraints in specific codon regions of the alignment at a particular branch of phylogenetic tree. The method estimates the null distribution of *d*_*S *_and *d*_*N *_from simulated sequence alignments. A statistically optimal window size is then estimated that makes the detection of adaptive evolution independent of the window size. Four codons was the estimated optimal window size. Simulated sequences were generated with the program EVOLVER from the PAML package [18] with parameters estimated from the true sequence alignments after running M0 codon-based model in CODEML.

### Identification of functionally important amino acid residues

To identify potentially functional and structural amino acids residues we used the Bayesian methods implemented in the ConSurf server [20]. Functionally important residues, e.g. involved in ligand binding and protein-protein interactions, are often evolutionarily conserved and are most likely to be solvent-accessible, whereas conserved residues within the protein core most probably have an important structural role in maintaining the protein properly folded [20].

### Secondary structure prediction and mutation mapping

Secondary structure prediction and tertiary fold-recognition (FR) were performed using the GENESILICO meta-server gateway [21]. Secondary structure was predicted using PSIPRED [22], PROFSEC [23], PROF [24], SABLE [25], and JNET [26]. FR methods did not report any good template structure for homology modeling procedure. Hence, *de novo *methods for structure modeling were applied.

### *De novo *structural modeling of TGBp3

To obtain a tridimensional structural model of the TGB3 protein we used TGBp3 sequences of P19, P22 and DB1 isolates. P19 and P22 represent necrotic and mild pathotypes of the CH2 genotype, respectively. DB1 isolates were described as necrotic isolates belonging to the EU genotype. The ROSETTA [27] algorithm was used for *de novo *modeling of TGBp3. Hundreds of thousands of decoys were generated and clustered to identify the best low-energy conformations. Selection of models based on the average energy clusters, size, density and visual inspection of final structures was performed. The quality of the final models was evaluated using the neural network based algorithm implemented in the PROQ server [28,29] and the MetaMQAPII meta-server [30]. In the first case, the quality is quantified by the location in the plane formed by the two indexes *LGscore *(i.e., the -log of a *p*-value) and *MaxSub *(ranging 0 - 1). Depending on the specific values of these indexes, the model can be qualified as: correct if *LGscore *> 1.5 and *MaxSub *> 0.1, as good if *LGscore *> 3 and *MaxSub *> 0.5, and as very good if *LGscore *> 5 and *MaxSub *> 0.8). Mapping of the electrostatic potential on protein surfaces was calculated with APBS (Adaptive Poisson-Boltzmann Solver) [31]. This procedure was performed to reveal potential electrostatic changes on the surface on the protein that may influence interaction between other proteins and elements in local cell environment.

## Results

### Analysis of TGBp3

The analysis of TGBp3 sequences reveled striking differences between particular isolates. TGBp3 consists of 246-258 nt and 82-86 amino acids, respectively, depending on the isolate considered. The protein of the Peruvian isolate LP-2001 from *Lycopersicon peruvianum *has two extra amino acids in the C-terminal region (86) than the characteristic 82-84 of other isolates. It is unclear whether this polymorphism in TGBp3 length has any affect on symptoms. The nucleotide identity between the 18 sequences studied ranged from 82% to 98.7% and from 77.3% to 98.7% at the amino acid level. The average transition/transversion rates ratio was high, 3.9, as observed for other plant RNA viruses [32].

**Figure 1:**
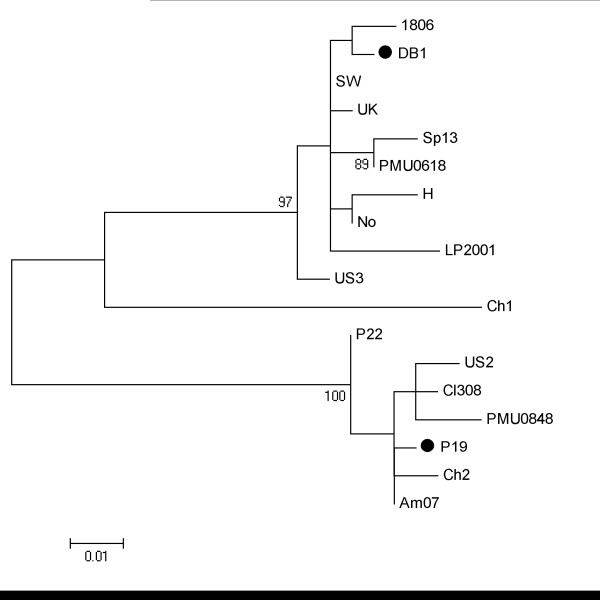
**Maximum-likelihood phylogenetic tree constructed from the nucleotide alignment of TGB3 sequences from the PepMV isolates listed in Table 1**. Numbers over the branches indicate the percentage of bootstrap support. Branches predicted to be under positive selection are indicated with black dots.

The phylogenetic tree shown in Figure 1 illustrates the presence of two major groups containing isolates from CH2 and EU genotypes. CH1 isolate form a separate branch. Both SLAC and REL method found no evidence of positively selected codons, but found three negatively selected ones: 50, 63 and 65 with *ω *= 0.36. By contrast, the FEL method predicted codon 37 as to be under positive selection, plus 15 other codons to be under purifying selection. Both CODEML and SWAPSC analyses revealed low *ω *value (0.35), thus suggesting a strong action of purifying selection in the evolution of TGB3 among the isolates studied. As mentioned above, *ω *< 1 is indicative of purifying selection removing deleterious nonsynonymous mutations. The whole TGBp3 has undergone purifying selection in the majority of branches of the phylogenetic tree. However, branch-site model implemented in CODEML showed positive selection acting in branches driving to necrotic isolates (Figure 1). In seven branches negative selection operated on region encompassing nucleotides 187-198 (amino acids 63-66), that includes the highly conserved PEVL motif. Those residues were also classified as functionally or structurally important by the Bayesian methods implemented in ConSurf. However, amino acid 67 (nucleotides 199-201) was predicted to be under positive selection, despite being surrounded by amino acids under negative selection. In this position, a K was found in the majority of PepMV isolates and only isolates DB1 (EU genotype) and P19 (CH2 genotype) contained E at this position, a very radical change replacing a basic residue by an acid one. Both isolates have been described as necrotic.

### Structural modeling of TGBp3

**Figure 2:**
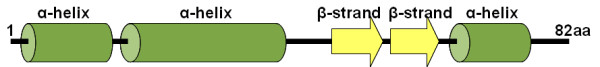
**Consensus results of the secondary structure prediction**. α-helices are show as green tubes and β-strands as yellow arrows.

Secondary structure prediction with PSIPRED [22] revealed the existence of three α-helices in regions 2-13, 17-36 and 62-68 and two β-strands in regions 45-50 and 53-57. Consistently, the modeled tridimensional structure preserved these structures. Position 67, which undergoes mutation K to E, is located in the third α-helix, without affecting the helix stability. A transmembrane domain/helix was predicted for the region encompassing amino acids 10 to 35 (Figure 2).

**Figure 3:**
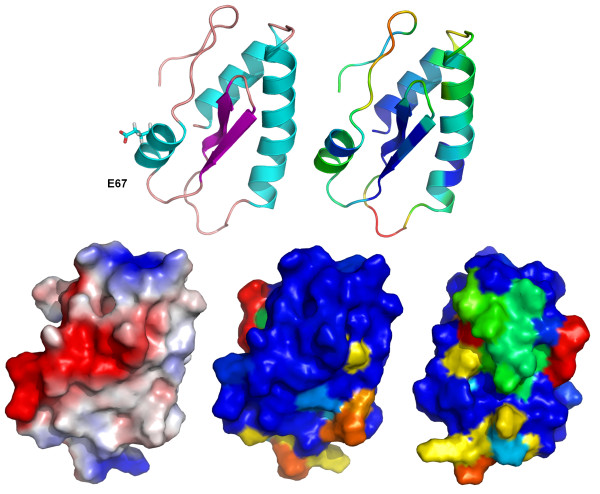
**Structural model of TGBp3**. The first panel shows a model in the ribbon representation colored according to the secondary structure elements. The second panel shows a model colored according to the predicted local deviation from the real structure, as calculated by METAMQAPII. Blue indicates low predicted deviation of C-α atoms down to 0 Å, red indicates unreliable regions with deviation > 5 Å, green to orange indicate intermediate values. The third panel shows a protein in the surface representation, colored according to the distribution of the electrostatic surface potential calculated with ABPS (see Methods). Blue indicates positively charged regions, red indicates negatively charged regions. The fourth and fifth panels show models in the surface representation, colored according to sequence similarity, calculated from multiple sequence alignments of PepMV sequences using ConSurf. All four models are in the same orientation, except the last one, which has been rotated horizontally. All figures have been drawn using PyMOL [44].

**Figure 4:**
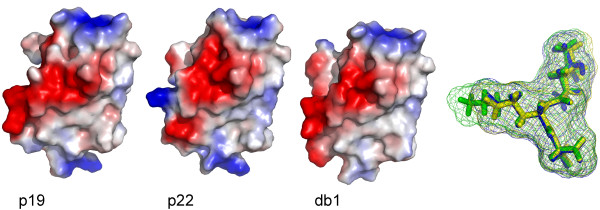
**Differences in mutated region for the TGBp3 for isolates P19, P22 and DB1**. First three proteins are in the surface representation, colored according to the distribution of the electrostatic surface potential calculated with ABPS (see Methods). Blue indicates positively charged regions, red indicates negatively charged regions. The fourth picture shows three superimposed regions - LEA for P19 (blue) and DB1 (yellow) isolates, LKA for P22 isolate (green). All figures have been drawn using PyMOL [44].

A three-dimensional model for the TGBp3 from the P19 necrotic isolate was obtained using the ROSETTA algorithm. The model was evaluated as "extremely good" according to the *LG score *(5.751) and "correct" according to the *MaxSub *index (0.320). The METAMQAPII method predicted that the overall GDT_TS score (Global Distance Test Total Score - a measure of similarity between two protein structures with identical amino acid sequences, but different tertiary structures) of the final model with respect to the native (currently unknown) structure is 59.45 (GDT_TS ranges between 1 and 100) and the model's root mean square deviation to the true structure is around 2.6. This indicates that the model can be used to make functional inferences at the level of individual residues. Overall, the inferred model for the TGBp3 (82 amino acids) consists of two antiparallel β-sheets flanked by three α-helices which exactly corresponds to those predicted by PSIPRED (Figure 3). Based on the structure of P19 isolate TGBp3, we modeled two other proteins: one from the mild isolate P22 and one from the necrotic isolate DB1. These two additional proteins were chosen to evaluate the effect of mutations conferring the necrotic phenotype on the protein structure. The amino acid sequence identity of TGBp3 between isolates P19 and DB1 was 85% and between P19 and P22 was as high as 99%. This level of amino acid identities allowed us to use homology-modeling methods [33] to build tridimensional models of both proteins. We particularly analyzed mutation K to E at residue 67. This mutation occurs rarely in PepMV homologous sequences. It is predicted to be in the surface of the folded protein and locally changing potential on the surface of helical region with mutation from negative to positive (Figure 4). This small change may influence on local interactions with environment components as well as the process of protein folding into native shape, but this needs further investigation.

## Discussion

Many plant virus genera encode a triple gene block (TGB), an especially evolutionarily conserved gene module involved in the cell-to-cell and long-distance movement of viruses. The TGB-based transport system exploits the coordinated action of three polypeptides to deliver viral genomes into plasmodesmata and to accomplish virus entry into neighboring cells [34,35]. TGB-encoded proteins are referred to as TGBp1, TGBp2 and TGBp3, according to the positions of their cistrons [35]. All three proteins are essential for virus movement. TGBp1 was widely studied and besides its role in movement, it has been shown that generally functions as an RNA silencing suppressor in members of the genus *Potexvirus *[36]. At the same time, little is know about additional function of TGBp2 and TGBp3. In agreement with sequence analysis and *in vitro *studies predicting that TGBp2 and TGBp3 are integral membrane proteins [37] cell fractionation of plant tissues expressing these proteins demonstrates predominant association of both proteins with the P1 and P30 membranous fractions as well as with the cell wall [38,39]. Understanding the molecular evolutionary biology of the various proteins expressed by viral genomes and their functions is a prerequisite for the control of virus propagation and the elaboration of efficient and durable antiviral strategies. In our previous study we have shown that TGBp3 is involved in host-pathogen interactions during PepMV infection. Experiments with PepMV TGBp3 mutants revealed that one single mutation K67E was required for converting a mild pathotype into a necrotic one [6]. Mutant viruses of mild PepMV strain induced necrosis on *Datura inoxia *and *Solanum lycopersicum*. Symptoms of viral infection strongly depended on the inoculated host plant and result from species-specific host-pathogen interactions.

Secondary and tertiary TGBp3 structure predictions showed that the protein consists of three α-helices and two β-strands. The secondary structure prediction placed amino acid 67 in one of the α-helices. Our tridimensional structural predictions revealed that the region encompassing amino acid 67 in isolates P19, P22 and DB1 is located on the surface of the protein and thus mutations in this region, specially when the physical properties dramatically change as it is the case of mutation K67E, must have a strong impact on the ability of TGBp3 to interact with other protein. It is well known that in many cases amino acids can be replaced without impairing protein function, even if these are of quite different physico-chemical characteristics. However, the change of a positively charged K by a negatively charged E may change the local property of the protein surface, jeopardizing its ability to establish the correct interactions with other viral proteins or cell components. Supporting the existence of such functionality in this surface region, we have identified nucleotide sites 187-198, spanning amino acids 63-66 that were under the action of negative selection in seven branches of the phylogenetic tree describing the evolutionary history of PepMV. In particular, the PEVL motif is highly conserved in all isolates analyzed.

In general, functionally essential protein parts are negatively selected for (conserved), while other parts can be positively selected for. The *ω *mean value obtained for the TGBp3 cistron strongly suggests a predominant action of purifying selection. In good agreement with this average value, three different approaches detected strong signal of purifying selection for particular codons. Nevertheless, most codons are evolutionarily neutral. However, perhaps the most interesting result from the analyses of selective constraints if that amino acid K67 has been identified as under positive selection in the branch leading to necrotic isolates, suggesting that necrosis may be an adaptive trait. Since it is close to the region of amino acids 63-66, which is (i) under negative selection (and hence likely has an important function) and (ii) predicted to be in the surface of the folded protein, we can speculate that it may be involved in the formation of protein-protein complex that determine the development of symptoms. The results obtained with ConSurf indicated that amino acids 61-66 played functional and structural roles in the protein. A protein function is however, the results of the functional and structural communication between sites and, therefore, the ability of a given site to change depends on the interactions it must establish with other residues of the molecule. Mutations at either nearby sites (like K67E), or functionally related distant sites in the structure, will change the selective constraints [40]. Functional sites, like binding domains, are less prone to amino acid changes than less important protein regions. Furthermore, some classes of proteins evolve faster than others [41].

The analysis of the TGB1 gene in PepMV populations clearly provides a mechanism for its rapid evolution and adaptation to the ever-changing environments [42]. In the light of what is so far known about PepMV evolutionary dynamics [43] it seems that TGBp3 evolves mostly by the action of purifying selection operating over several sites, highlighting its functional role during PepMV infection. Gómez et al. [43] showed only one amino acid under purifying selection in the TGBp3 of CH2 genotype also using the methods implemented in the DATAMONKEY server. We have analyzed TGBp3 sequences representing different genotypes and we used more sophisticated methods to establish selection pressure acting on TGBp3. We were able to identify more codons under action of purifying selection, moreover amino acid 67 was predicted to be under positive selection. It seems that the particular pathotypes achieved an advantage over others especially in Europe. Recently, more aggressive pathotypes (causing necrosis or severe yellowing) of CH2 genotype have become dominant in Poland. This shift in the Polish PepMV population reveals a dynamic interplay between the different PepMV genotypes and their host. Necrotic isolates, however, were described in both the EU and CH2 genotypes, and it seems that the selective pressures act in the direction of increasing the virulence of isolates, less temperature dependent which cause significant losses in quality and quantity of yield. Preliminary data (Hasiów-Jaroszewska, unpublished results) have shown that necrotic isolates acquired the ability to infect a larger number of *Solanum tuberosum *varieties and of causing more severe symptoms in shorter time. Probably, the key feature of these isolates are faster replication or accumulation. It seems that molecular evolution is leading to higher variability among CH2 genotype in comparison to others genotypes. It has been suggested that the CH2 genotype has a biological advantage over the EU genotype, as it seems to spread more rapidly within a crop [5]. Recent study on the evolutionary dynamics of the PepMV population in Spain using RT-quantitative PCR analyses in inoculated tomato plants showed that a CH2 isolate (PS5) accumulated more rapidly and to higher viral loads than an EU isolate (Sp13) [43]. The TGBp3 is responsible for virus movement and further research will be performed to shed the light between particular mutation in TGBp3 and virus fitness.

The present *in silico *study opens new research avenues for researches interested in experimentally exploring the *in vivo *interactions between TGBp3 and host factors. Moreover, we report the first tridimensional structure of TGBp3, obtained with *de novo *folding methods followed by careful accuracy assessments. This model may serve as a platform for further sequence, structural and function analysis and will stimulate new experiment advances.

## Conclusions

The obtained results suggested that TGBp3 has been evolving mainly under the action of purifying selection operating on several amino acid sites however amino acid 67, which has been previously shown to be a necrosis determinant, was found to be under positive selection. The estimated tridimensional structure of TGBp3 will serve as a platform for further sequence, structural and function analysis and will stimulate new experimental advances.

## Competing interests

The authors declare that they have no competing interests.

## Authors' contributions

BHJ and SFE participated in the design of the study. BHJ and AC performed analyses and drafted the manuscript. SFE and HP participated in drafting the final version of manuscript. All authors read and approved the final manuscript.
